# Artificial Intelligence for Medicines Information: Scoping Review of Clinical Applications and Digital Health Inequalities

**DOI:** 10.2196/77747

**Published:** 2026-03-06

**Authors:** Shahd Al-Arkee, Josephine Falade, Vibhu Paudyal

**Affiliations:** 1 Department of Life Sciences, University of Bath, Cleaverton Down, Bath, BA2 7AY, United Kingdom, 44 1225383782; 2 Research Department of Practice and Policy, UCL School of Pharmacy, University College London, London, United Kingdom; 3 Department of Health Services and Population Research, King’s College London, London, United Kingdom

**Keywords:** artificial intelligence, medicines information, pharmacy, digital health inequalities, scoping review

## Abstract

**Background:**

Artificial intelligence (AI) has the potential to support medicines information services. However, a comprehensive mapping of its use, particularly within pharmacy practice and in the context of digital health inequalities, is lacking.

**Objective:**

This scoping review mapped existing evidence on AI-driven medicines information, focusing on the accuracy and completeness of AI-generated content, the role of health care professionals (HCPs), particularly pharmacists, and the impact of digital health inequalities on AI adoption.

**Methods:**

This scoping review was informed by the methodological framework proposed by Levac et al, which includes modifications to the original Arksey and O’Malley scoping review framework. A systematic search was conducted across MEDLINE (Ovid), PubMed Central, Cochrane Library, CINAHL Plus (EBSCOhost), International Pharmaceutical Abstracts (IPA), Web of Science, and Google Scholar from inception to January 2025, which served as the search cutoff date. Peer-reviewed studies in English evaluating the role of AI in medicines information across any health care settings (including patient homes) were included. The results are reported in accordance with the PRISMA-ScR (Preferred Reporting Items for Systematic Reviews and Meta-Analyses extension for Scoping Reviews) guidelines.

**Results:**

A total of 1911 citations were identified, with 14 studies meeting the inclusion criteria. AI tools showed promise in supporting medicines information services but were found to have limitations in accuracy, particularly when applied to complex clinical queries. Pharmacists were the most engaged HCPs in the evaluation of AI-generated content. Only 3 studies explored digital health inequalities in the context of AI and access to medicines information. Reported barriers included misinformation risks, regulatory gaps, and digital health inequalities, particularly infrastructure limitations and disparities in digital literacy, which affected AI adoption.

**Conclusions:**

AI-driven tools show promise in supporting medicines information services, but concerns remain. HCPs, particularly pharmacists, play a critical role in AI evaluation and validation, yet their involvement remains ill-defined. Addressing digital health inequalities is essential for effective AI integration. Future research should focus on identifying and minimizing digital health inequalities, as well as evidence-informed AI implementation in medicines information services.

## Introduction

Artificial intelligence (AI), often described as “computational intelligence” or the “science and engineering of creating intelligent machines” [1], is a rapidly evolving field focused on replicating human-like behavior in computers and related technologies [2]. A key subset of AI, machine learning (ML), enables computers to analyze vast datasets and improve predictive accuracy without explicit programming [3]. AI applications in health care range from rule-based decision systems to advanced ML tools. By identifying patterns and learning from experience, ML algorithms can assist in predicting patient outcomes and supporting clinical decision-making across various health care settings [4].

AI has recently demonstrated increasing potential in pharmacy practice, particularly in medication management. A recent systematic review analyzing multiple AI algorithms reported that technology-driven approaches can enhance medication management in primary care [5]. The growing integration of AI into health care has led to the development of various AI interventions, including algorithms designed to support both patients and health care professionals (HCPs) in disease management [6]. Several reviews have evaluated AI beyond medicines information, encompassing interventions in disease management [7], diagnosis [8], and shared decision-making [9]. Other reviews highlighted AI’s role in pharmacy practice, demonstrating its effectiveness in optimizing medication management, enhancing drug safety, and improving clinical decision support systems [10]. However, the application of AI specifically to medicines information remains underexplored. Medicines information is recognized as a key component of promoting rational use of medicines, ensuring prescribers, dispensers, and consumers have access to independent and unbiased information about medication use [11].

Despite the growing interest in AI applications, there remains a need to comprehensively map how AI is used in medicines information services. Existing research lacks a clear synthesis of applications of AI, the accuracy and completeness of AI-generated responses, and the challenges influencing its adoption. Pharmacists and other HCPs play a crucial role in evaluating and integrating AI-driven tools, yet the extent of their engagement and its implications for pharmacy practice remain unclear. Furthermore, digital health inequalities refer to disparities in access to and use of digital health technologies, which are often shaped by factors such as internet connectivity and geographic location. These may influence the equitable implementation of AI across diverse health care settings.

This scoping review aims to map the current landscape of AI in medicines information, focusing on its applications, content accuracy and completeness, and adoption within pharmacy practice.

## Methods

### Information Sources and Search Strategy

This scoping review was conducted following the PRISMA-ScR (Preferred Reporting Items for Systematic Reviews and Meta-Analyses extension for Scoping Reviews) guidelines [12], available in Checklist 1. The methodological framework proposed by Levac et al [13], which expands on the original approach by Arksey and O’Malley [14], was used to guide the review. The protocol was developed in accordance with the following steps that are (1) identifying the research questions; (2) identifying relevant studies; (3) selecting studies through a team-based approach for study selection and data extraction; (4) charting the data using a structured approach; and (5) collating, summarizing, and reporting the results. This review was guided by four research questions that are (1) What impact do AI tools have on the dissemination and accessibility of medicines information in different health care settings, particularly in pharmacy practice? (2) What is the reported accuracy and completeness of AI-generated medicines information, and what factors influence its reliability? (3) What are the barriers and facilitators to AI adoption, particularly in addressing digital health inequalities? (4) How do HCPs, particularly pharmacists, perceive and engage with AI tools for medicines information? This protocol was registered and available on the Open Science Framework website [15].

A systematic literature search was conducted across MEDLINE (Ovid), PubMed Central, Cochrane Library, CINAHL Plus (EBSCOhost), International Pharmaceutical Abstracts (IPA), Web of Science, and Google Scholar from inception to January 2025. The database searches were conducted between January 10 and 20, 2025, with January 31, 2025, set as the inclusion cutoff date. A 4-domain search strategy was used, including terms related to AI, medicines information, digital health inequalities, and the role of the pharmacy workforce, particularly pharmacists, in AI adoption. The search strategy was developed and refined by 2 reviewers in consultation with an expert from the University College London Pharmacy School library. The final search strategy is presented in Multimedia Appendix 1 and was formatted in line with PRESS (Peer Review of Electronic Search Strategies) guidelines [16].

### Study Selection and Data Extraction

Studies were included if they were qualitative, quantitative, or mixed methods studies, published in English in peer-reviewed journals, and explored the role of AI in enhancing medicines information. Medicines information was defined as a key component of promoting rational use of medicines, ensuring prescribers, dispensers, and consumers have access to independent and unbiased information about medication use [11] and guided the eligibility assessment. Eligible studies involved AI-enabled tools, including rule-based expert systems and ML models. Tools lacking any AI or ML functionality, such as conventional digital databases, were excluded. Reviews, editorials, commentaries, letters, media data, gray literature (eg, reports, theses, and white papers), and conference abstracts were also excluded to ensure methodological rigor and consistency.

A 2-stage selection process was used. In the first stage, one reviewer (SA) screened titles and abstracts for relevance and removed duplicate records, and the second reviewer (VP) thoroughly assessed the titles and abstracts for inclusion. In the second stage, 2 reviewers independently evaluated the full-text studies against the prespecified eligibility criteria. Disagreements were resolved through discussion and consensus. Bibliographies of selected studies were manually searched to identify additional relevant references.

Data extraction was performed using a standardized form developed specifically for this review. Extracted data included study characteristics, AI intervention details, and key findings related to AI-generated medicines information.

### Data Synthesis and Quality Assessment

Data were synthesized using a descriptive approach, following a narrative synthesis method. The evidence was mapped to the predefined categories of HCP involvement, accuracy and completeness of AI-generated content, and implications for digital health inequalities. Study characteristics were also summarized, and gaps in the literature were highlighted. A formal quality assessment of the included studies was not conducted, in line with Arksey and O’Malley methodological framework for scoping reviews [14]. However, variations in study quality and methodological limitations were described narratively to support transparency and interpretation.

## Results

### Search Results and Study Selection

**Figure 1.**
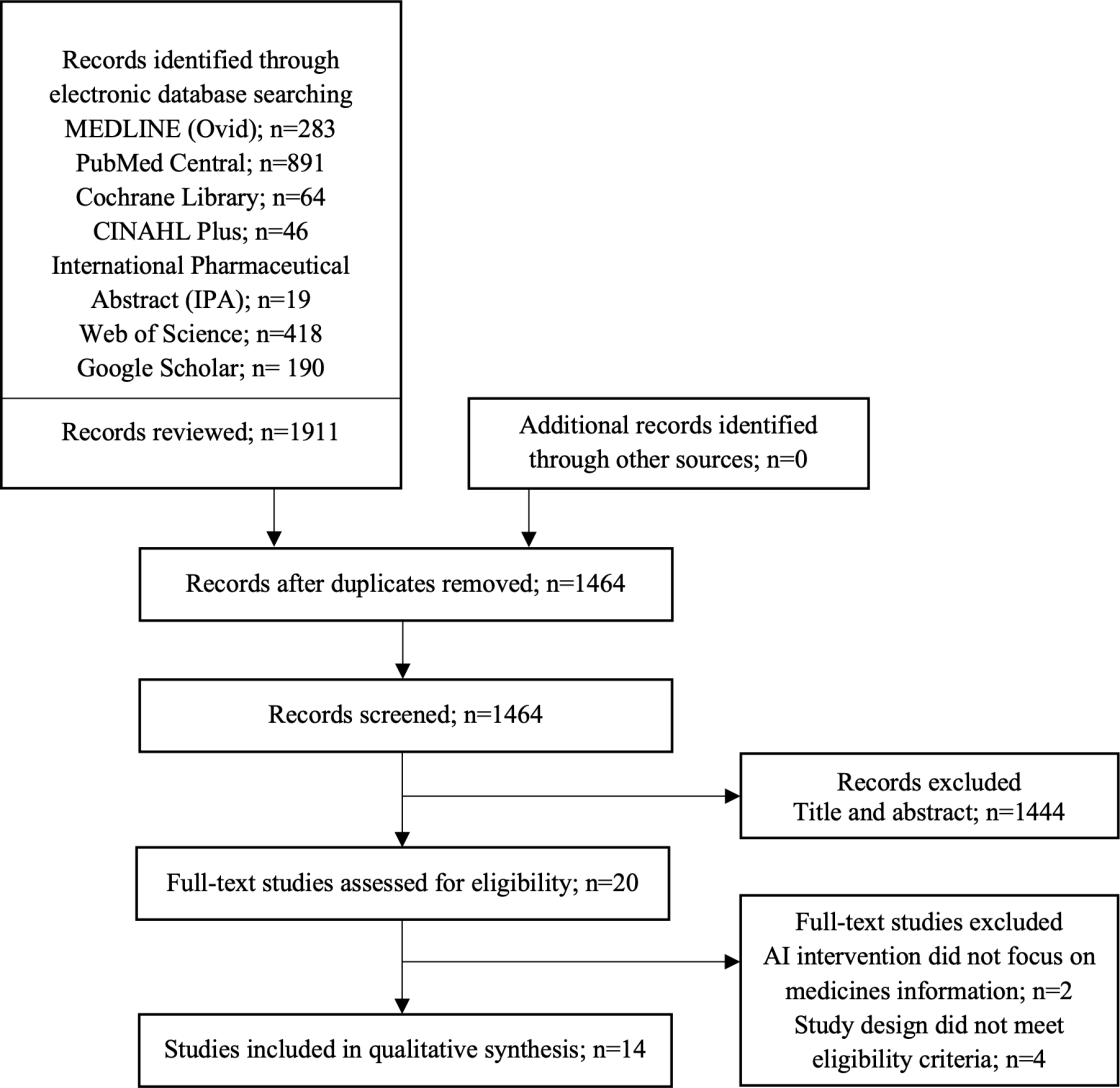
PRISMA (Preferred Reporting Items for Systematic Reviews and Meta-Analyses) flow diagram depicting study selection. AI: artificial intelligence.

The systematic search yielded 1911 citations, of which 447 duplicates were removed, resulting in 1464 records screened by title and abstract. Of these, 20 studies were assessed for full-text review. Six studies were excluded, and no additional records were identified through manual searching. Therefore, 14 studies met the inclusion criteria and were included in this review [17-30]. A PRISMA (Preferred Reporting Items for Systematic Reviews and Meta-Analyses) flowchart summarizing the study selection process is shown in Figure 1.

### Study Characteristics and Design

The included studies were published between 2021 and 2025. Most originated from high-income economies, as classified by the World Bank, including Australia [26], Canada [21], Germany [17], the Netherlands [30], Singapore [27], and the United States [18-20,22,25,29]. In contrast, only a few studies originated from middle- and low-income countries, such as Egypt [28], Ethiopia [24], and India [23]. All studies focused on the application of AI for delivering medicines information (Table 1).

**Table 1.** Characteristics of the included studies.

Study; country	Health care setting	Data sample size	Research purpose	Reported outcomes
Andrikyan et al [17]; Germany	Online patient drug information search	500 chatbot responses (readability, completeness, and accuracy), 20 responses (safety)	Assess chatbot-generated drug advice for quality, readability, and safety concerns	Readability Completeness Accuracy Safety
Beavers et al [18]; United States	Inpatient hospital	200 real-world medication-use questions	Analyze the clinical completeness, correctness, usefulness, and safety of chatbot and medication database responses to everyday inpatient medication-use questions	Clinical correctness Completeness Safety
Cornelison et al [19]; United States	AI^a^-driven medication counseling	240 chatbot responses	Evaluate chatbot accuracy and completeness in answering medication-related patient questions	Accuracy Completeness
Grossman et al [20]; United States	Academic drug information service	39 medication-related questions	Evaluate ChatGPT’s accuracy, completeness, and relevance in drug inquiries	Accuracy Completeness Satisfactory Performance by question complexity
Laymouna et al [21]; Canada	Pharmacists in HIV care (community and hospital)	41 pharmacists from 15 municipalities	Assess pharmacists’ knowledge, attitudes, and practices in HIV care and evaluate the usability of an AI-based chatbot for medicines information support	Pharmacists’ knowledge, attitudes, practices, and perceived barriers Usability of the chatbot
Munir et al [22]; United States	Clinical pharmacy practice	32 pharmacy-based clinical questions	Assess ChatGPT’s accuracy in answering pharmacy-related clinical queries	Accuracy Case-based questions
Ramasubramanian et al [23]; India	Not stated	462 medication dosage-related queries	Evaluate the accuracy of ChatGPT 3.5, ChatGPT 4, and Google Bard in providing medical drug dosages based on Harrison’s Principles of Internal Medicine	Accuracy
Sendekie et al [24]; Ethiopia	Community pharmacies	225 pharmacists	Investigate pharmacists’ perceptions and willingness to use AI in practices, and their perceived barriers to its implementation	Perception of AI and willingness to use AI Barriers to AI adoption
Sheikh et al [25]; United States	Nephrology and pharmacy practice	124 nonprescription drugs and supplements assessed	Evaluate ChatGPT in drug safety for patients with kidney disease	Accuracy Safety
Stanceski et al [26]; Australia	Hospital discharge summaries (MIMIC-IV database)	100 chatbot responses (summaries)	Evaluate responses for safety, accuracy, and language simplification for AI-generated patient-centered discharge instructions	Accuracy Language simplification
Sumner et al [27]; Singapore	Outpatient clinics (hospital)	20 (10 health care providers, 10 patients)	Develop and evaluate an AI-driven nudge intervention for medication adherence	Acceptance Preference and concerns
Taha et al [28]; Egypt	Pharmacy practice (community and hospital pharmacies)	428 pharmacists	Explore pharmacists’ perceptions, practices, and concerns regarding ChatGPT in pharmacy practice	Perceptions on accuracy Privacy concerns
Triplett et al [29]; United States	Academic-based drug information center	84 ChatGPT responses analyzed	Assess the accuracy, completeness, and consistency of ChatGPT responses to drug information inquiries compared to drug information center responses	Accuracy Readability
van Nuland et al [30]; The Netherlands	Hospital-based clinical pharmacy practice	30 clinical pharmacy questions	Evaluate ChatGPT’s ability to provide appropriate responses to clinical pharmacy questions and assess accuracy and consistency	Accuracy Completeness

a AI: artificial intelligence.

The included studies used a range of study designs. Eight studies used comparative and evaluation methodologies to assess AI-generated medicines information accuracy and usability [18-20,22,23,25,26,29]. Two studies used cross-sectional survey designs to explore user perceptions, knowledge, and attitudes toward AI-based tools [17,28]. Two studies adopted a human-centered design approach to assess AI-driven interventions: one evaluating AI-generated patient discharge instructions [26], and another developing an AI-driven nudge tool for medication adherence [27]. While most studies used quantitative research approaches**,** 2 studies used survey-based needs assessment designs to explore contextual factors and AI implementation considerations [21,24]. The studies were conducted across diverse health care settings, including community-based and primary care [21,24,28], secondary care (outpatients) [22,26,27], tertiary care settings [18,25,30], and web-based patient communities (online platforms without direct clinical engagement) [17,19,20,29]. One study did not specify a health care setting [23].

### AI Intervention Characteristics

The AI tools evaluated across the included studies showed diverse functionalities in delivering medicines information (Table 2). Most AI systems used natural language processing (NLP) to generate medication-related responses [17-20,22,23]. Microsoft Bing Copilot analyzed user queries and provided structured drug information, while Micromedex with Watson generated text-based responses but struggled with complex clinical scenarios [17,18]. ChatGPT was widely assessed using various versions, with studies evaluating its accuracy in answering patient and academic drug inquiries, pharmacy-based clinical questions, and drug dosage recommendations [19,20,22,23]; among these, ChatGPT-4 (OpenAI) outperformed other models in a comparative evaluation [23].

Other included studies focused more on AI applications, adoption barriers, or decision-making, rather than primarily evaluating NLP-generated responses [21,24-30]. The MARVIN-Pharma chatbot supported pharmacists in HIV care, enhancing access to adherence counseling and clinical guidelines [21]. An evaluation of AI adoption in community pharmacies revealed that while AI could streamline medication management, infrastructure, and training barriers limited its potential [24]. Another study explored the utility of AI in nephrology and pharmacy practice, finding that ChatGPT sometimes misclassified supplement safety, thereby indicating the need for human oversight [25]. In hospital settings, AI-generated discharge summaries improved readability but also raised safety concerns [26]. AI-driven nudges enhanced medication adherence through personalized reminders for outpatients [27]. While AI tools improved efficiency and medication counseling, concerns persisted regarding misinformation, privacy, and patient-specific recommendations [28]. AI-generated drug information improved clarity but required pharmacist oversight, and hospital pharmacy AI responses often lacked reliability, raising concerns about clinical decision-making [29,30].

**Table 2.** Characteristics of the AI^a^ interventions.

Study; country	AI name and functionality	AI potential risk	Research conclusion
Andrikyan et al [17]; Germany	Microsoft Bing Copilot: for patient drug-related queries	Incomplete or inaccurate drug information, and readability issues	Capable of providing complete and accurate information Not reliable for medical advice without validation
Beavers et al [18]; United States	Micromedex with Watson (IBM Watson Health): AI chatbot integrated with an evidence-based pharmacological knowledge base (MDX), using natural language processing to provide accurate, complete, and safe responses to medication-use questions	Limited scope, incomplete answers, and potential safety concerns	Traditional drug databases outperformed AI chatbots in completeness, safety, and usefulness Chatbot responses were clinically correct but not always complete or safe
Cornelison et al [19]; United States	ChatGPT-3.5: general AI chatbot for patient medication-related questions	Incomplete or inaccurate drug information	Mostly accurate for answering common medication-related questions May provide incomplete or inaccurate information
Grossman et al [20]; United States	ChatGPT 3.5: general AI chatbot for pharmaceutical medication-related questions	Incomplete or inaccurate drug information and fabricated references	Unreliable for medication-related inquiries due to frequent inaccuracies, incomplete responses, and misleading references
Laymouna et al [21];Canada	MARVIN-Pharma: AI chatbot designed to support pharmacists in delivering evidence-based medicines information for HIV care	Usability concerns, with pharmacist engagement required	AI could support HIV care Needs pharmacist engagement in the chatbot’s development
Munir et al [22]; United States	ChatGPT 3.5: generative AI language model designed to answer clinical questions in pharmacy practice	Misleading information, poor performance in complex cases, and safety concerns	Limited success Excels in calculations Struggles with drug information and patient-specific cases
Ramasubramanian et al [23]; India	ChatGPT 3.5, ChatGPT 4, Google Bard: AI models assessed for drug dosage accuracy, based on Harrison’s Principles of Internal Medicine	Accuracy varied across diseases and organ systems	ChatGPT 4 exhibited the highest accuracy Requires further validation before clinical application
Sendekie et al [24]; Ethiopia	Not specified: generic AI applications in pharmacy: for personalized patient care and decision support	Needs for resources, policies, training, and infrastructure	AI is recognized as beneficial Needs training and policy development for successful integration
Sheikh et al [25]; United States	ChatGPT 3.5 and ChatGPT 4: evaluated for assessing the safety of nonprescription medications and supplements in kidney disease, compared to Micromedex	Inaccuracy, lack of reliability, and patient safety concerns	ChatGPT 4 has potential, but remains unreliable compared to established databases Further AI development is needed before clinical adoption
Stanceski et al [26]; Australia	ChatGPT 3.5 (via Microsoft Azure OpenAI): AI-generated patient-friendly discharge instructions from hospital summaries	Potential medication errors	AI tools can generate patient-centered discharge instructions Require clinician oversight before implementation
Sumner et al [27]; Singapore	AI-driven nudge system for medication adherence, integrating reminders, social references, and automated interventions	Digital literacy barriers for older adults, and data privacy concerns	Promising for improving medication adherence but must be tailored to user needs Needs flexible delivery, simplified data entry, and caregiver involvement
Taha et al [28]; Egypt	ChatGPT: AI-driven drug information tool assisting pharmacists with counseling, drug interactions, and clinical queries	Concerns on accuracy, data privacy, and bias	Potential applications in pharmacy but requires accuracy improvements and regulatory guidelines
Triplett et al [29]; United States	ChatGPT 3.5: AI-generated responses for pharmaceutical inquiries, based on NLP^b^ and public databases	Accuracy concerns, lack of references, requiring pharmacist validation	ChatGPT provided clear and readable responses Lacked accuracy, requiring expert review for reliability
van Nuland et al [30]; The Netherlands	ChatGPT 3.5: AI-generated responses for hospital pharmacy inquiries, including dosing, drug interactions, and therapeutic drug monitoring	Lack of response accuracy, inconsistent AI-generated information, risk of medication errors	ChatGPT offered clinical advice Inconsistent accuracy and reproducibility, making it unsuitable for independent clinical use

a AI: artificial intelligence.

b NLP: natural language processing.

### Accuracy and Completeness

Accuracy was primarily evaluated based on the correctness of AI-generated responses in providing medication-related information, but only 5 studies [22,23,25,26,29] conducted a direct assessment. A study evaluated ChatGPT-3.5, ChatGPT-4, and Google Bard for drug dosage recommendations. ChatGPT-4 demonstrated the highest performance (83.77%), but accuracy varied across diseases and organ systems [23]. Another study assessing AI’s classification of drug safety in patients with kidney disease found that ChatGPT-4 had higher agreement (81.4%) with Micromedex than ChatGPT-3.5 (64.5%), yet it remained unreliable for independent clinical use [25]. Similarly, a study analyzing AI-generated discharge summaries found that 18% of responses contained potentially harmful safety issues, including 6% with “hallucinated information,” meaning responses that appeared plausible but were actually inaccurate and 3% introducing unprescribed medications [26]. Another study compared ChatGPT’s accuracy in drug information inquiries against a drug information center, reporting an accuracy rate of 50%. ChatGPT lacked proper references and reliability for clinical use [29]. Another study identified significant limitations in ChatGPT’s accuracy, particularly in patient-specific scenarios, emphasizing the need for verification before integration into pharmacy practice [22].

Five additional studies evaluated both accuracy and completeness, evaluating how well AI responses covered all essential information. Microsoft Bing Copilot achieved 100% median accuracy and completeness across 500 responses, yet experts reviewing a subset of 20 chatbot responses found that 66% were potentially harmful, with 22% classified as life-threatening [17]. Micromedex with Watson produced 85% clinically correct responses, but only 65% were complete and 71% acceptable for safety [18]. AI-generated responses to hospital pharmacy inquiries were only 26% correct and complete, while 22% were correct but incomplete, 30% partially correct, and 22% completely incorrect [30]. One study found ChatGPT-generated responses were 92.5% accurate and 80.8% complete, though 4.2% contained minor errors [19]. Another study reported that only 26% of ChatGPT’s responses were satisfactory, while 38% lacked a direct response, 38% contained inaccuracies, and 41% lacked completeness [20]. A detailed grouped summary of all included studies by AI task and evaluation metric is presented in Table 3.

**Table 3.** Grouped summary by AI^a^ task and metric.

AI task	Study; country	Evaluation metric	Outcome
Drug information	Andrikyan et al [17]; Germany	Accuracy Safety	100% median accuracy 66% potentially harmful (subset safety analysis)
	Beavers et al [18]; United States	Accuracy Completeness Safety	85% accuracy 65% completeness 71% safety
	Cornelison et al [19]; United States	Accuracy Completeness	92.5% accuracy 80.8% completeness
	Grossman et al [20]; United States	Accuracy Completeness	26% satisfactory Among unsatisfactory responses, 38% lacked accuracy and 41% lacked completeness
	Taha et al [28]; Egypt	Perception Concerns	Perceived benefits (73.6%) Concerns about privacy, bias, and accuracy
	Triplett et al [29]; United States	Accuracy Reference Quality	50% accuracy Poor reference quality
	van Nuland et al [30]; The Netherlands	Accuracy Completeness	26% correct and complete 22% correct but incomplete
Dosage recommendations	Ramasubramanian et al [23]; India	Accuracy	83.77% accuracy Performance varied by disease
HIV care	Laymouna et al [21]; Canada	Perception Usability	Perceived potential to support HIV care, pharmacist engagement essential
Clinical queries	Munir et al [22]; United States	Expert judgment	Good in calculation (100%) Poor in complex cases (20%) Safety concerns
System-level use	Sendekie et al [24]; Ethiopia	Infrastructure readiness Perception	Positive perception Policy and training needed for integration
Safety assessment	Sheikh et al [25]; United States	Agreement Safety	81.4% agreement (ChatGPT-4) with Micromedex Not yet reliable for clinical use
Discharge instructions	Stanceski et al [26]; Australia	Safety	18% potentially harmful Incorrect medication content
Adherence support	Sumner et al [27]; Singapore	Perception	Acceptable and promising User-centered design emphasized

a AI: artificial intelligence.

### Digital Health Inequalities

Aspects of digital health inequalities investigated within the included studies related to AI-driven interventions to enhance health care access, support medication adherence, and provide drug information. Key barriers identified were limited infrastructure and digital literacy (Table 4).

**Table 4.** Summary of AI^a^ adoption barriers by country context.

Study; country	Research setting (geography)	Barriers category	Key barriers identified	Patient-level inequities
Sendekie et al [24]; Ethiopia	LMIC^b^	Structural or institutional	Lack of internet Lack of AI tools Limited training	Infrastructure Digital literacy
Sumner et al [27]; Singapore	HIC^c^	Personal	Digital literacy among older adults Privacy concerns	Age Data privacy concerns
Taha et al [28]; Egypt	LMIC	Personal or demographic	Regional gaps in awareness and training Limited accuracy of AI Data privacy	Geographic disparities Low income and education levels

a AI: artificial intelligence.

b LMIC: low- and middle-income country.

c HIC: high-income country.

AI-driven medication adherence tools showed promise, with one study demonstrating improved adherence through personalized reminders and automated interventions, but digital literacy barriers among older adults and privacy concerns limited their effectiveness [27]. Infrastructure and accessibility challenges were key barriers to AI adoption. A study in Ethiopia found that 89.3% of surveyed pharmacists cited lack of internet availability, 88.2% lacked AI-related software and hardware, and 80.9% reported insufficient training. More than 90% emphasized the need for structured policies, better internet access, and AI-focused training to support AI integration in pharmacy services [24].

Regional disparities in AI awareness and adoption were evident. A study evaluating Egyptian pharmacists’ perceptions of ChatGPT found that those in Greater Cairo showed the highest level of AI awareness, whereas those in South Upper Egypt had the lowest, reflecting geographic variations in AI exposure. Limited AI training further hindered equitable adoption and impacted AI-supported decision-making. Concerns about AI accuracy, data privacy, and bias reinforced skepticism toward AI as a standalone clinical tool, emphasizing the need for expert validation in pharmacy practice [28].

### Involvement of HCPs

The involvement of HCPs in AI interventions varied across the included studies, with pharmacists being the primary group engaged. This aligns with the focus of our scoping review on their role in AI-driven medicines information. Five studies specifically involved pharmacists, either as primary users of AI [20,29] or as evaluators of AI-generated responses [22,25,30]. Three studies explored pharmacists’ perceptions of AI, assessing their willingness to integrate AI into practice, perceived barriers to adoption, and concerns regarding accuracy, data privacy, and trust in AI-based recommendations [21,24,28]. These studies provided valuable insights into pharmacists’ readiness to adopt AI-driven health care solutions while highlighting challenges related to infrastructure, regulatory frameworks, and professional training.

Four studies included multidisciplinary HCPs, such as physicians and nurses, in evaluating and validating AI interventions [17,18,26,27]. In contrast, 2 studies reported no direct involvement of HCPs in AI use or evaluation, as AI-generated responses were independently assessed by reviewers whose professional backgrounds were not specified [19,23].

### Narrative Appraisal of Included Studies

Several studies relied on small or narrowly defined datasets [22,25], hypothetical scenarios without clinical validation [17,26], or educational and nonclinical settings [19,20,24,29], which limited their real-world applicability. Evaluations were frequently conducted internally, without independent or blinded assessors [21,30], and some lacked comparisons to human experts or assessments of clinical consequences [22,23]. In addition, poor reproducibility [30], limited topic scope [25], single-center study design [18], and nonsystematic prompt design were apparent [27,28].

## Discussion

### Principal Findings

This scoping review provided an overview of AI-driven tools in medicines information, focusing on HCP involvement, AI accuracy and completeness, and digital health inequalities. Some models accurately address general medication inquiries but struggle with complex clinical questions, raising concerns regarding reliability, data privacy, and usability. With respect to HCP involvement, studies highlighted the multidisciplinary engagement, particularly in medication adherence and discharge planning. Pharmacists played a key role in AI evaluation and integration. Regarding equity, the review highlighted disparities in infrastructure, digital literacy, and access to technology, which may hinder equitable implementation. One study examined digital health inequalities from the patient perspective.

Although a formal quality appraisal was not conducted, several methodological limitations were observed across the included studies, which should be considered when interpreting the overall strength and applicability of the evidence.

### Comparison With Prior Work

The included studies presented AI interventions used for medicines information, particularly in their potential applications in medication inquiries, adherence monitoring, and discharge planning. These findings align with previous reviews, which have explored the capacity of AI to support medication adherence through predictive models and real-time monitoring. AI-driven tools, including ML-driven monitoring systems, have been investigated for their ability to assess adherence patterns and identify at-risk patients; however, their effectiveness varies based on implementation and patient engagement [31]. Similarly, ML and statistical models have been explored for their potential in optimizing discharge planning by predicting patient outcomes, which may help reduce readmission rates and improve hospital efficiency [32]. Despite these promising applications, challenges remain regarding the accuracy, data privacy, and usability of AI-generated outputs. A recent systematic review highlighted that while AI has been explored in various aspects of medication use, its application in supporting clinical decision-making and complex, patient-specific inquiries remains limited [33]. Although AI models often demonstrate high accuracy when handling standard medication-related questions, their performance tends to decline in more complex clinical contexts, reinforcing the importance of human oversight. This review builds on these findings by specifically examining how AI is being applied to support medicines information services and evaluating the quality and focus of these emerging interventions.

The successful integration of AI into medication management and the provision of medicines information requires the active involvement of HCPs, particularly pharmacists, physicians, and nurses. The included studies indicate that pharmacists are key stakeholders in the adoption, evaluation, validation, and clinical implementation of AI tools. Similarly, a systematic review highlighted the potential of AI to enhance decision-making, emphasizing the need for expert validation to ensure clinical relevance and safety [34]. Furthermore, multidisciplinary engagement is essential for successful AI adoption; involving HCPs in AI development and validation improves usability and fosters trust. A narrative review identified key factors influencing trust in medical AI—such as explainability, transparency, and usability—reinforcing the importance of clinician involvement in ensuring effective decision-making [35].

Challenges remain in AI implementation, particularly in relation to digital health inequalities. Some included studies highlighted regional disparities in AI adoption, with pharmacists working in urban areas showing greater familiarity with AI tools compared to their counterparts working in rural settings [24,28]. While these studies provide valuable insights into pharmacists’ access to and perceptions of digital tools, only one study specifically addressed digital health barriers from the patient perspective [27]. This highlights a key research gap in understanding how AI-driven pharmacy interventions affect patients, particularly vulnerable populations. Furthermore, limited AI-related training and infrastructure barriers, particularly in low-resource settings, hinder AI adoption [24], whereas the study from a high-income country emphasized individual-level concerns [27]. These findings align with broader research highlighting technological gaps and digital literacy disparities as significant barriers to AI adoption in medicines information services [36].

A key challenge in AI-generated medicines information involves potential risks related to data privacy, regulatory gaps, and ethical concerns. Two included studies reported inaccuracies or inconsistencies in AI-generated responses, raising concerns about their reliability and implications for patient safety [23,25]. This aligns with findings from a previous narrative review on AI governance, which highlighted the need for clear regulatory frameworks to support the adoption of AI in medicines information, particularly in addressing issues of bias, transparency, and accountability [37].

### Strengths and Limitations

To the best of the authors’ knowledge, this is the first scoping review to comprehensively map the use of AI for medicines information and evaluate its accuracy. A key strength is its systematic approach, adherence to established scoping review methodology, incorporating both qualitative and quantitative study designs.

Certain limitations should be acknowledged. The exclusion of gray literature, while consistent with our protocol and justified by the need to prioritize methodological rigor, may have introduced publication bias. Given the fast-evolving nature of AI research, new AI applications may be reported initially in non–peer-reviewed sources. Future updates of this review may benefit from a broader inclusion strategy or a targeted sensitivity search of these sources. Other limitations relate to the heterogeneity of included studies. The limited number of evaluated AI tools, small sample size, and small number of countries and within specific health care settings restrict the ability to draw generalizable conclusions. Inconsistencies were observed in how included studies defined and measured key outcomes such as “accuracy” and “completeness.” Some studies assessed accuracy based on comparison to expert responses, while others used subjective scoring or evaluated only surface-level correctness. Similarly, the concept of “completeness” varied, ranging from the presence of keywords to the inclusion of all critical information. This variability complicates direct comparisons across studies and limits the ability to synthesize consistent conclusions regarding AI performance. Moreover, some application areas remain underexplored. Importantly, no formal quality appraisal of the included studies was performed, which may affect the ability to assess the robustness of individual findings—although this is consistent with established scoping review methodology.

### Implications for Pharmacy Practice

This review highlights pharmacists as the most actively engaged HCPs in the development, evaluation, and implementation of AI tools for medicines information. Their clinical expertise, accessibility, and experience in medication counseling position them as key stakeholders in ensuring the safe and effective use of AI-driven systems. Pharmacists require targeted training to critically evaluate AI-generated information, understand algorithmic limitations, and interpret outputs in clinical context. Embedding digital health and AI literacy into pharmacy education and continuing professional development will be critical to building confidence and capability in using these tools. Particular attention should be paid to pharmacists working in low-resource settings who lack access to digital health technologies.

Workflow integration also requires attention. AI systems should be incorporated seamlessly into pharmacy practice to enhance efficiency without disrupting established routines. This may involve embedding AI within electronic health records, clinical decision support systems, and patient counseling processes. Co-designing tools with pharmacists can ensure alignment with routine tasks and improve adoption.

Policy and governance considerations are equally important. Pharmacists should contribute actively to shaping standards for validation, data privacy, and accountability, especially in patient-facing contexts. Interdisciplinary collaborations will be essential as medicines information becomes increasingly digitized. Pharmacists are well-positioned to lead interdisciplinary teams in the development, use, and appraisal of AI applications and the information they generate.

### Implication for Research

This review provides an overview of AI’s role in medicines information; however, several gaps remain that warrant further investigation. Future research should focus on improving the accuracy and reliability of AI in handling complex clinical inquiries, as current models demonstrate inconsistencies in delivering clinically relevant recommendations. Equally important is the evaluation of the real-world effectiveness of AI in supporting medication adherence and discharge planning through personalized interventions.

Research should also assess the impact of digital health inequalities on AI adoption. In this review, only a small subset of studies explicitly examined aspects such as disparities in access to AI tools, language inclusivity, or representation of vulnerable populations. This limited evidence base restricts our ability to draw conclusions about the equity implications of AI-generated medicines information. Given that AI systems can perpetuate or even exacerbate existing health care disparities, future research should assess how factors such as access to digital technologies, digital literacy, and systemic bias influence access to and trust in AI tools, particularly in underresourced settings. Particular attention needs to be paid to inequalities that intersect with digital literacy skills, including older age, socioeconomic disadvantage, and people living in remote and rural areas. Frameworks such as the Digital Health Equity Framework [38] or the PROGRESS-Plus (Place of residence, Race/ethnicity/culture/language, Occupation, Gender/sex, Religion, Education, Socioeconomic status, and Social capital) lens [39] can provide conceptual guidance for equity-centered investigations. Future research should also explore ways to facilitate accessibility, affordability, and digital literacy skills from the perspectives of HCPs who practice in low-resource settings.

While this review identified pharmacists as key stakeholders in AI evaluation, further studies are needed to assess how AI influences clinical decision-making, workflow integration, and patient communication across various health care settings. Equally important is the integration of patient perspectives in the design and evaluation of AI tools. Despite their critical role as end users, patients were largely underrepresented in the included studies, indicating a need for more patient-centered research to ensure AI solutions are usable, trustworthy, and aligned with patient needs.

### Conclusions

This scoping review highlights the early promise of AI in supporting medicines information, but its reliability, especially in complex clinical scenarios, remains uncertain. At present, AI should be viewed as a complementary tool rather than a replacement for human expertise, with HCPs playing a critical role in its evaluation and integration. Key barriers such as digital health inequalities, ethical concerns, and regulatory gaps must be addressed to enable the safe and equitable implementation. Future research should focus on identifying and mitigating digital health inequalities in this context, including intersectional disadvantages as well as improving evidence-based use of AI, expanding training for HCPs, and developing robust governance frameworks to support standardized AI integration into clinical practice.

## Supplementary material

10.2196/77747 Multimedia Appendix 1 Search strategy (PRESS) Format.

10.2196/77747 Checklist 1 PRISMA-ScR guidelines.

## Abbreviations

AI: artificial intelligence
HCP: health care professional
IPA: International Pharmaceutical Abstracts
ML: machine learning
NLP: natural language processing
PRESS: Peer Review of Electronic Search Strategies
PRISMA: Preferred Reporting Items for Systematic Reviews and Meta-Analyses
PRISMA-ScR: Preferred Reporting Items for Systematic Reviews and Meta-Analyses Extension for Scoping Reviews
PROGRESS-Plus: Place of residence, Race/ethnicity/culture/language, Occupation, Gender/sex, Religion, Education, Socioeconomic status, and Social capital
