# Supplementary material for: Artificial Intelligence for Medicines Information: Scoping Review of Clinical Applications and Digital Health Inequalities
Source: J Med Internet Res. 2026 Mar 6;28:e77747. doi: 10.2196/77747 (PMC12978933; doi:10.2196/77747)
Supplement: Multimedia Appendix 1 [file jmir-v28-e77747-s001.pdf]

Multimedia Appendix 1. Search Strategy (PRESS Format)

Ovid MEDLINE (1946 to January 2025; 283 records retrieved)

1. exp Artificial Intelligence/
2. exp Machine Learning/
3. Neural Networks*.mp.
4. (Artificial Intelligence* or Machine Learning* or Neural Network*).mp.
5. ((Deep Learning or Autonomous Systems or AI) adj3 tools).mp.
6. (Intelligent Systems or Intelligent Algorithms or Algorithmic Learning).mp.
7. 1 or 2 or 3 or 4 or 5 or 6
8. Drug Information Services/
9. Medicines Information*.mp.
10. (Medicines Information or Drug Information or Medication Dissemination).mp.
11. 8 or 9 or 10
12. (Accuracy or Verification or Reliability).mp.
13. ((Accurate adj3 information) or (Reliable adj3 information)).mp.
14. 12 or 13
15. exp Health Disparities/
16. Digital Health Inequalities.mp.
17. ((Digital Divide or digital health) adj3 inequalities).mp.
18. 15 or 16 or 17
19. exp Pharmaceutical Services/
20. ((Pharmacy adj3 Counsel) or (Pharmacist Intervention)).mp.
21. (Pharmacy Workforce or Pharmacy Capacity).mp.
22. 19 or 20 or 21
23. 7 and 11
24. 7 and 11 and 14
25. 7 and 18
26. 7 and 11 and 22
27. 23 or 24 or 25 or 26

PubMed Central (inception to January 2025; 891 records retrieved)

(("Artificial Intelligence"[MeSH Terms] OR "Machine Learning"[MeSH Terms] OR "AI tools"[Title/Abstract]) AND ("Drug Information Services"[MeSH Terms] OR "Medicines Information"[Title/Abstract] OR "dissemination"[Title/Abstract]))

OR

(("Artificial Intelligence"[MeSH Terms] OR "Machine Learning"[MeSH Terms] OR "AI-driven technologies"[Title/Abstract]) AND ("Drug Information"[Title/Abstract] OR "Medicines Information"[Title/Abstract]) AND ("Accuracy"[Title/Abstract] OR "Verification"[Title/Abstract] OR "Reliability"[Title/Abstract]))

OR

(("Artificial Intelligence"[MeSH Terms] OR "Machine Learning"[MeSH Terms] OR "AI tools"[Title/Abstract]) AND ("Digital Health Inequalities"[Title/Abstract] OR "Digital Divide"[Title/Abstract]))

OR

(("Artificial Intelligence"[MeSH Terms] OR "Machine Learning"[MeSH Terms] OR "AI tools"[Title/Abstract]) AND ("Pharmacy Workforce"[Title/Abstract] OR "Workforce Capacity"[Title/Abstract] OR "Pharmacists"[MeSH Terms]))

Cochrane Library (inception to January 2025; 64 records retrieved)

"Artificial Intelligence" OR "Machine Learning"

AND "medicines information" OR "drug information"

AND "pharmacy practice" OR "pharmacists"

In Title Abstract Keyword

A broad Title/Abstract /Keyword strategy was used to maximise sensitivity in the Cochrane Library.

Google Scholar (Inception to January 2025; 190 records retrieved)

"Artificial Intelligence" AND ("medicines information" OR "drug information")

A broad search strategy was used in Google Scholar, reflecting the platform’s limited Boolean functionality. During title and abstract screening, additional eligibility concepts were applied, including relevance to pharmacy practice or pharmacists, accuracy or reliability of medicines information, and/or digital health inequalities, consistent with scoping review methodology.

CINAHL Plus (EBSCOhost) (Inception – January 2025; 46 records retrieved)

S1 (MH "Artificial Intelligence") OR (MH "Machine Learning") OR (MH "Neural Networks") OR
TI ("Artificial Intelligence*" OR "Machine Learning*" OR "Neural Networks*") OR
AB ("Artificial Intelligence*" OR "Machine Learning*" OR "Neural Networks*") OR
TX ("Deep Learning" OR "Autonomous Systems" OR "AI tools" OR "Intelligent Systems" OR "Intelligent Algorithms" OR "Algorithmic Learning") OR
TX ("AI" N3 "tools")

S2 (MH "Drug Information") OR
TX ("Medicines Information*" OR "Medication Dissemination")

S3 TX ("Accuracy" OR "Verification" OR "Reliability") OR
TX ("Accurate" N3 "information") OR
TX ("Reliable" N3 "information")

S4 (MH "Health Disparities") OR
TX ("Digital Divide") OR
TX ("Digital Health" N3 "Inequalities")

S5 (MH "Pharmaceutical Services") OR
TX ("pharmacy" N3 "counsel*") OR
TX ("Pharmacist Intervention") OR
TX ("Pharmacy Workforce") OR
TX ("Pharmacy Capacity")

S6 S1 AND S2
S7 S1 AND S2 AND S3
S8 S1 AND S4
S9 S1 AND S2 AND S5
S10 S6 OR S7 OR S8 OR S9

Web of Science (inception to January 2025; 418 records retrieved)

#1 ALL=("Artificial Intelligence*" OR "Machine Learning*" OR "Neural Networks*" OR "Deep Learning" OR "Autonomous Systems" OR "AI tools" OR "Intelligent Systems" OR "Algorithmic Learning" OR "Intelligent Algorithms")

#2 ALL=("Medicines Information*" OR "Drug Information*" OR "Medication Dissemination")

#3 ALL=("Accuracy" OR "Verification" OR "Reliability")

#4 ALL=("Digital Divide" OR "Digital Health Inequalities" OR "Healthcare Inequalities")

#5 ALL=("Pharmacy Counsel" OR "Pharmacist Intervention" OR "Pharmacy Workforce" OR "Pharmacy Capacity")

#6 #1 AND #2
#7 #1 AND #2 AND #3
#8 #1 AND #4
#9 #1 AND #5
#10 #6 OR #7 OR #8 OR #9

International Pharmaceutical Abstract (IPA) (inception to January 2025; 19 records retrieved)

1. (AI OR "Artificial Intelligence*" OR "Machine Learning*" OR "Neural Networks*" OR "Deep Learning" OR "Autonomous Systems" OR "AI tools" OR "Intelligent Systems" OR "Algorithmic Learning").mp.
2. ("Medicines Information*" OR "Drug Information*" OR "Medication Dissemination").mp.
3. ("Accuracy" OR "Verification" OR "Reliability" OR "Accurate Information" OR "Reliable Information").mp.
4. ("Digital Divide" OR "Digital Health Inequalities").mp.
5. ("Pharmacy Counsel" OR "Pharmacist Intervention" OR "Pharmacy Workforce" OR "Pharmacy Capacity").mp.
6. 1 AND 2
7. 1 AND 2 AND 3
8. 1 AND 4
9. 1 AND 5
10. 6 OR 7 OR 8 OR 9
